# Pathophysiological Mechanisms of Myocardial Bridging-Related Angina and Ischemia with Implications for Therapeutic Strategies

**DOI:** 10.3390/cells15100888

**Published:** 2026-05-13

**Authors:** Srdjan Aleksandric, Barry Uretsky, Ana Djordjevic-Dikic, Dejan Orlic, Nebojsa Antonijevic, Milorad Tesic, Stefan Juricic, Marko Banovic, Vojislav Giga, Nikola Boskovic, Zlatko Mehmedbegovic, Ivana Jovanovic, Dejan Simeunovic, Sinisa Stojkovic, Vladan Vukcevic, Goran Stankovic, Branko Beleslin

**Affiliations:** 1 Cardiology Clinic, University Clinical Center of Serbia, Koste Todorovica 8, 11000 Belgrade, Serbia; skali.ana7@gmail.com (A.D.-D.); orlicmail@yahoo.com (D.O.); drantoni@gmail.com (N.A.); misa.tesic@gmail.com (M.T.); stefan.juricic@gmail.com (S.J.); markobanovic71@gmail.com (M.B.); voja2011@yahoo.com (V.G.); belkan87@gmail.com (N.B.); zlatkombegovic@gmail.com (Z.M.); dejan.simeunovic@mfub.bg.ac.rs (D.S.); sstojkovi@open.telekom.rs (S.S.); vladan.vukcevic@gmail.com (V.V.); gorastan@gmail.com (G.S.); branko.beleslin@gmail.com (B.B.); 2 Faculty of Medicine, University of Belgrade, 11000 Belgrade, Serbia; 3 Central Arkansas Veterans Health System, Little Rock, AR 72205, USA; barry.uretsky@va.gov; 4 Department of Medicine, Division of Cardiology, University of Arkansas for Medical Sciences, Little Rock, AR 72205, USA

**Keywords:** myocardial bridging, angina, myocardial ischemia, coronary vasospasm, atherosclerosis, coronary microvascular dysfunction, coronary functional testing, treatment

## Abstract

**Highlights:**

**What are the main findings?**

- Myocardial bridging may cause anginal symptoms and/or myocardial ischemia through several different pathophysiological and cellular mechanisms acting independently or synergistically.
- The main coronary flow disturbances in a coronary artery with myocardial bridging are: (1) the presence of characteristic diastolic coronary flow velocity profile (diastolic “finger-tip” phenomenon) within and distal to the myocardial bridging; (2) the presence of retrograde coronary flow in the arterial segment proximal to the myocardial bridging during systole; and (3) increased diastolic/systolic velocity ratio within and distal to the myocardial bridging.

**What are the implications of the main findings?**

- Lifelong coronary flow disturbances in coronary artery with myocardial bridging lead to: (1) changes in flow-generated endothelial (wall) shear stress; (2) morphological and functional changes in both endothelial and vessel smooth muscle cells; and (3) changes in the expression of both atheroprotective and proatherogenic molecules which differ proximally, within, and distally to the bridge.
- Comprehensive coronary physiology testing should be encouraged in patients with this coronary anomaly to identify the underlying cause of anginal symptoms and/or myocardial ischemia, enabling optimal therapeutic strategies in these patients.

**Abstract:**

Myocardial bridging (MB) is a congenital coronary anomaly characterized by systolic compression of the intramyocardial arterial segment and delayed early diastolic artery relaxation, resulting in reduced vessel luminal diameter in diastole. Current evidence suggests that MB, particularly in the left anterior descending artery, may cause anginal symptoms and/or myocardial ischemia through several different pathophysiological and cellular mechanisms acting independently or synergistically: (1) delayed early diastolic relaxation of intramyocardial arterial segment; (2) impaired endothelial-dependent vasodilation with vessel smooth muscle cell hyperactivity in the coronary artery with MB, especially within the bridged segment; (3) focal (septal) ischemia due to “septal steal” phenomenon; and (4) development and progression of an atherosclerotic lesion in the coronary artery segment proximal to MB. Patients with isolated-MB may also experience anginal pain and/or myocardial ischemia due to concomitant structural and/or functional abnormalities of the coronary microcirculation. Both MB and coronary microvascular dysfunction refer to a subgroup of patients with angina and/or ischemia with non-obstructive coronary arteries (ANOCA/INOCA). Therefore, it may be challenging to determine whether MB is causing anginal pain and/or ischemia, particularly since both phenomena have also been reported without MB’s existence. Therefore, comprehensive coronary physiology testing should be encouraged in patients with this coronary anomaly to identify the underlying cause of anginal pain and/or myocardial ischemia, enabling optimal therapeutic strategies in these patients. This review is focused on different pathophysiological and cellular mechanisms of MB-related angina and/or ischemia and future perspectives in the functional assessment of MB severity, bearing in mind the complexity of coronary physiology in the presence of this anomaly.

## 1. Introduction

**Figure 1:**
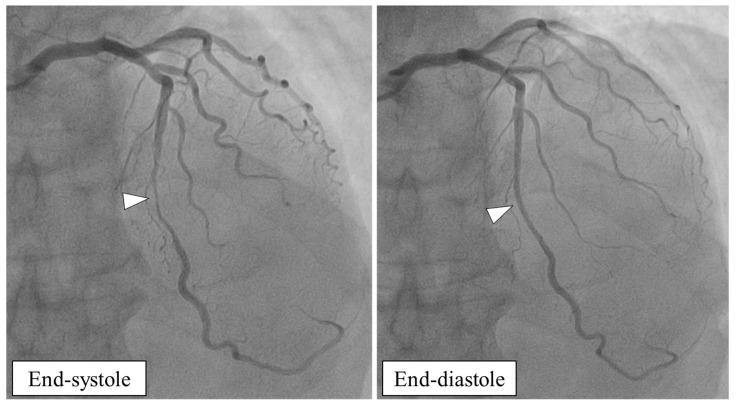
Invasive coronary angiography of a patient with isolated myocardial bridging (MB: arrow head) in the medial segment of the left anterior descending coronary artery (LAD). Note systolic compression of the intramyocardial LAD segment (“milking effect”) with reduced vessel luminal diameter at end-diastole. MB-patient with inducible myocardial ischemia on stress echocardiography and diastolic fractional flow reserve (d-FFR) ≤ 0.76.

Myocardial bridging (MB) is a congenital coronary anomaly with an intramyocardial arterial course (referred to as an “intramyocardial arterial segment” or “tunneled artery”) that is constricted by the overlying myocardial tissue (referred to as a “myocardial bridge”) [1,2,3,4,5,6]. It is characterized by systolic compression of the intramyocardial arterial segment on invasive coronary angiography (so-called “milking effect”) and delayed early diastolic artery relaxation, resulting in reduced vessel luminal diameter in diastole (Figure 1) [7,8,9,10]. This so-called dynamic stenosis is usually observed in the left anterior descending (LAD) artery (>90% of cases) [6]. Autopsy incidence for LAD ranges from 15 to 85%, while coronary computed tomography angiography (CCTA) incidence ranges from 30 to 50% [11,12,13,14,15,16,17,18,19,20,21,22,23,24,25,26,27,28,29,30,31,32,33,34,35,36,37,38]. On invasive coronary angiography (ICA), however, MB on LAD has been reported between 0.5 and 14%, meaning most of these MBs do not result in angiographically visible systolic compression [31,39,40,41,42,43,44,45,46,47,48,49,50,51,52,53]. This is explained by the different CCTA-based anatomical and morphological characteristics of MB located on the LAD determined by its depth and anatomical course relative to the interventricular septum and the right ventricle’s (RV) anterior wall (Table 1) [21].

Myocardial bridging is generally considered a benign anatomic variation, but when located in the LAD, it may cause myocardial ischemia, acute coronary syndrome, life-threatening ventricular arrhythmias, left ventricle (LV) systolic dysfunction, or even sudden cardiac death (SCD) [1,2,3,4,5,6,7,8,9,10,54,55]. Although it is a congenital coronary anomaly, it was observed that clinical symptoms and objective signs of myocardial ischemia in patients with isolated-MB on the LAD (without associated atherosclerotic obstructive coronary artery disease [CAD]) usually appear after the fourth or fifth decade of life [3,5,55]. When and why a previously asymptomatic patient with a congenital MB on the LAD becomes symptomatic and/or associated with myocardial ischemia is poorly understood.

**Figure 2:**
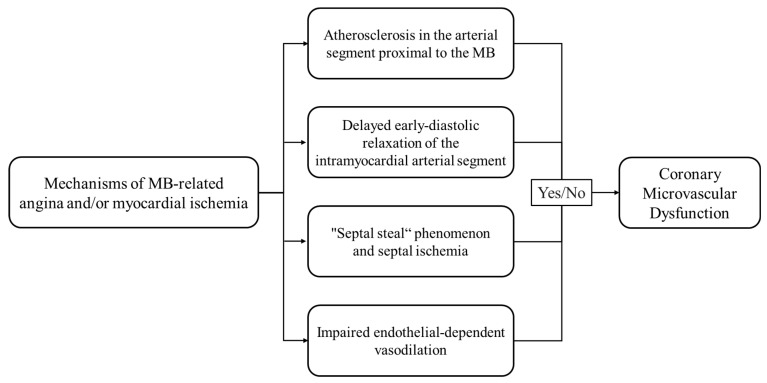
Angina and/or myocardial ischemia in patients with myocardial bridging (MB) and non-obstructive coronary artery disease can arise from various pathophysiologic mechanisms, which may act independently or synergistically. Patients with MB may also experience angina and/or myocardial ischemia due to concomitant structural and/or functional abnormalities of the coronary microcirculation.

The occurrence of severe cardiac events has raised the question of the most appropriate test/s that could be helpful in the identification of MB associated with myocardial ischemia. However, the clinical, hemodynamic, and prognostic significance of this anomaly is still undetermined, as different revascularization strategies failed to produce the expected benefit due to the high incidence of complications [4,5]. Several studies showed that percutaneous coronary intervention (PCI) of “symptomatic” and angiographically significant isolated-MB on the LAD, defined as systolic MB-compression ≥ 50% diameter stenosis (DS), was associated with a high rate of in-stent restenosis, regardless of the stent type, and with a higher rate of coronary perforation and stent fracture with subsequent thrombosis [56,57,58,59,60,61,62,63,64,65,66]. Similarly, surgical treatment involving either supra-arterial myotomy (“unroofing”) or coronary artery bypass grafting (CABG) was also associated with high rates of RV perforation, LV aneurysm formation, and graft failure [67,68,69,70,71,72,73,74,75]. In this regard, identifying an MB associated with anginal symptoms and/or myocardial ischemia is essential to avoid unnecessary interventions. Current evidence suggests that MB, particularly in the LAD, may cause anginal symptoms and/or myocardial ischemia through several different pathophysiological and cellular mechanisms acting independently or synergistically: (1) delayed early diastolic relaxation of intramyocardial arterial segment; (2) focal (septal) ischemia due to “septal steal” phenomenon; (3) impaired endothelial-dependent vasodilation with vessel smooth muscle cell hyperactivity (VSMCs) in the coronary artery with MB, especially within the bridged segment; and (4) development and progression of atherosclerotic lesion in the coronary artery segment proximal to MB (Figure 2) [1,2,3,4,5,6,7,8,9,10,54,55,76,77].

Patients with isolated-MB may also experience anginal pain and/or myocardial ischemia due to concomitant structural and/or functional abnormalities of the coronary microcirculation (Figure 2). Both MB and coronary microvascular dysfunction (CMD) refer to a subgroup of patients with angina and/or ischemia with non-obstructive coronary arteries (ANOCA/INOCA) [78,79,80]. Therefore, comprehensive coronary physiology testing should be encouraged in patients with this coronary anomaly to identify the underlying cause of anginal pain and/or myocardial ischemia, enabling optimal therapeutic strategies in these patients. This review is focused on different pathophysiological and cellular mechanisms of MB-related angina and/or ischemia and future perspectives in the functional assessment of MB severity, bearing in mind the complexity of coronary physiology in the presence of this anomaly.

## 2. Myocardial Bridging and Myocardial Ischemia

A well-established explanation for exertional ischemia in MB patients is inadequate diastolic relaxation of the intramyocardial arterial segment, resulting in limited coronary blood flow [8,10,54,55,81,82]. It was previously believed that the functional significance of isolated-MB on the LAD was predominantly determined by the degree of systolic compression of its intramyocardial segment [39,46,50,53]. Although a positive correlation exists between MB-depth and systolic MB-compression at ICA, there is no correlation between MB-depth and anginal symptoms or objective signs of inducible ischemia, according to many previously published studies [8,9,10,54,55,81]. Furthermore, no study has demonstrated a significant correlation between systolic MB-compression and anginal symptoms and/or inducible ischemia [8,9,10,54,55,82]. These studies also confirmed that anginal symptoms and/or objective signs of myocardial ischemia in MB patients were closely correlated with the percent DS at late-diastole, but not at systole. In the invasive hemodynamic study using quantitative coronary angiography (QCA) in combination with conventional and diastolic fractional flow reserve (FFR and d-FFR, respectively) measurements, the percent DS of intramyocardial LAD segment at late-diastole was one of the independent predictors of stress-induced myocardial ischemia in MB patients [8]. This is in line with the fact that coronary blood flow occurs primarily during diastole (approximately 80% to 85%), especially in the LAD. Previous studies with intravascular ultrasound (IVUS) and/or QCA have also found that systolic compression of the intramyocardial LAD segment persists during early diastole due to delayed relaxation with subsequent persistent diastolic diameter reduction during mid-to-late diastole [8,54,55,83,84,85,86,87]. According to these studies, the average diameter reduction in mid-to-late diastole was between 29% and 41% DS at the MB-site, exceeding 50% DS in 10% of MB patients. These findings suggest that diastolic decompression of the intramyocardial LAD segment is probably slow and delayed, and followed by incomplete vessel diameter gain during mid-to-late diastole. This phenomenon is known as “delayed early diastolic relaxation” of the intramyocardial LAD segment, which may worsen by rapid atrial pacing, inotropic provocation with dobutamine, or exercise stress testing due to tachycardia and shortening of diastolic perfusion (filling) time [8,9,10,54,55,82,85].

**Figure 3:**
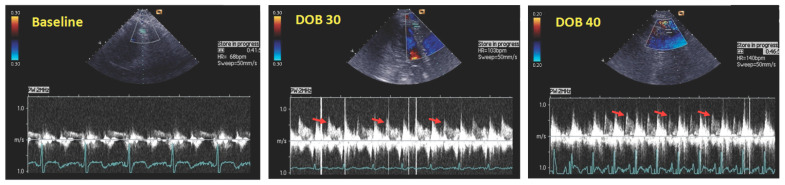
Characteristic diastolic coronary flow velocity profile (“finger-tip” phenomenon: red arrows) in the distal segment of left anterior descending artery, below the myocardial bridging, obtained by transthoracic Doppler echocardiography during high-dose dobutamine infusion (DOB: 30–40 µg/kg/min). This phenomenon is characterized by an abrupt coronary flow acceleration followed by rapid deceleration during early diastole and mid-to-late diastolic flow plateau. Adapted with permission from Aleksandric et al. [54].

Studies measuring coronary flow velocity at rest and during steady-state hyperemia with 0.014-inches of intracoronary Doppler wires throughout the cardiac cycle at the MB-site, as well as in LAD segments just proximal and distal to the MB, revealed that this coronary anomaly may produce significant hemodynamic changes in coronary flow: (1) the presence of characteristic diastolic coronary flow velocity profile (diastolic “finger-tip” phenomenon) at the MB-site, as well as in the LAD segment distal to the MB; (2) the presence of retrograde coronary flow during systole in the LAD segment proximal to the MB; and (3) increased diastolic/systolic velocity ratio (DSVR) [55,83,84,85,86,87,88]. The characteristic diastolic coronary flow velocity profile is characterized by an abrupt coronary flow acceleration during early diastole, followed by a rapid deceleration during mid-diastole and mid-to-late diastolic flow plateau (Figure 3). This diastolic “finger-tip” phenomenon was also confirmed by the study using non-invasive transthoracic Doppler echocardiography (TTDE) to measure resting and hyperemic diastolic coronary flow velocity and coronary flow reserve (CFR) in MB patients, which was further augmented during dobutamine provocation (Figure 3) [54].

During early diastole, ventricular relaxation occurs, resulting in a rapid reduction in microcirculatory resistance and intracoronary pressure at the distal end of the coronary artery [89,90]. It induces an immediate increase in coronary perfusion pressure followed by an immediate increase in diastolic coronary flow velocity [89,90]. In normal coronary arteries, this results in a gradual acceleration of coronary flow during early diastole, followed by a gradual decline until the beginning of systole. Patients with obstructive CAD have a similar coronary flow velocity profile, but with a reduced hyperemic coronary flow velocity response and reduced CFR [91,92]. Conversely, the rapid increase in early diastolic coronary flow velocity in the intramyocardial LAD segment and distal artery is the result of combined maximal coronary perfusion pressure and persistent diameter reduction, resulting in a steep diastolic pressure gradient across the MB (Figure 3) [54,55,83,84,85,86,87,88]. The following lumen gain at mid-diastole rapidly reduces this pressure gradient and diastolic coronary flow velocity, which reaches its plateau during mid-to-late diastole, when the diameter of the intramyocardial LAD segment is at its maximum (Figure 3) [54,55,83,84,85,86,87,88]. Therefore, the major coronary flow disturbance in the LAD with MB occurs during early-to-mid diastole, when coronary flow and myocardial perfusion are at the highest level. However, even in the absence of a coronary lesion, this early diastolic myocardial perfusion is the highest in the subepicardium, while subendocardial perfusion is delayed by 10 to 20 s and reaches only 45% of the maximal early diastolic subepicardial perfusion [2,89,93]. Early diastolic coronary flow and myocardial perfusion will remain preserved as long as diastolic relaxation (decompression) of the intramyocardial LAD segment is preserved. In the presence of significantly delayed early diastolic relaxation of the intramyocardial LAD segment, coronary flow within and distal to the MB will be impaired, and subendocardial perfusion below the ischemic threshold may occur during atrial pacing, dobutamine, or exercise stress testing, which simultaneously reduces diastolic perfusion time and increases LV pressure due to tachycardia and increased LV oxygen demand [2,89,93]. Accordingly, subendocardial ischemia in patients with isolated-MB on the LAD is a result of an interplay between: (1) delayed early diastolic relaxation of the intramyocardial LAD segment, which may further deteriorate during exercise- or dobutamine-induced tachycardia; (2) reduced diastolic perfusion time; and (3) inhibited early diastolic coronary flow with consequent subendocardial hypoperfusion. In addition, LV diastolic dysfunction associated with aging and LV hypertrophy may unmask or aggravate MB-associated angina and/or ischemia due to its negative effects on early diastolic MB relaxation and diastolic perfusion time [2,3].

## 3. Myocardial Bridging and “Septal Steal” Phenomenon

**Figure 4:**
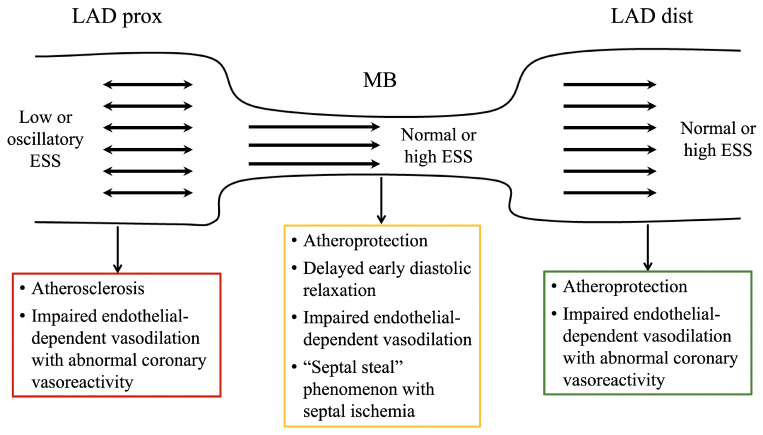
A schematic illustration of the left anterior descending artery (LAD) with myocardial bridging (MB) and varying endothelial shear stress (ESS) values across the artery, along with the potential mechanisms of MB-related angina and/or myocardial ischemia in different LAD segments. Low and oscillatory ESS simultaneously increases proatherogenic agent production and decreases atheroprotective agent production in the proximal LAD segment (LAD prox). Conversely, normal or high ESS has an atheroprotective effect by upregulating atheroprotective molecules and downregulating proatherogenic molecules in the intramyocardial and distal LAD segments (LAD dist).

Patients with MB on the LAD, regardless of the MB-depth, may present with atypical or angina-like chest pain, but without typical objective signs of inducible ischemia, as a result of focal (septal) ischemia due to “septal steal” or “branch steal” phenomenon [2,89,94,95]. A study by Lin et al. found that anginal symptoms in MB patients without “typical” apex or anterior wall motion abnormalities (WMA) were caused by focal ischemia in the vascularization zone of one or more septal branches originating from the intramyocardial LAD segment and were presented as focal septal WMA with apical sparing on exercise stress echocardiography [95]. They revealed that focal ischemia is a consequence of a large increase in coronary flow velocity at early diastole as it passes through the narrowed intramyocardial LAD segment (tunneled segment), causing a large pressure drop at the MB-site with, consequently, an abrupt decrease in perfusion pressure in septal branches, which leads to septal ischemia (“septal steel” phenomenon) (Figure 2 and Figure 4). This so-called “Venturi” effect relies on Bernoulli’s principle and the principle of conservation of energy, according to which the product of fluid velocity and pressure must be equal at all points of a closed tube. According to these findings, at least two questions emerge: (1) it is unclear whether this focal (septal) ischemia seen in MB patients is clinically significant, considering that non-invasive stress tests cannot demonstrate typical objective signs of moderate-to-severe ischemia; and (2) data regarding the prognostic value of this focal (septal) ischemia is still unclear [96,97,98].

## 4. Myocardial Bridging and Atherosclerosis

Studies with intracoronary Doppler tracings also discovered decreased or absent systolic antegrade coronary flow in the intramyocardial LAD segment, as well as in the LAD segment distal to the MB [55,84,85,86,87,88]. Due to increased early diastolic flow and reduced systolic flow, the DSVR within and distal to the MB is significantly higher in comparison to normal coronary arteries [55,84,85,86,87,88]. Moreover, it was found that systolic retrograde coronary flow may occur toward the LAD segment proximal to the MB during this phase, which may be further enhanced by nitroglycerin or dobutamine due to their positive inotropic and chronotropic effects on the myocardium [55,84,85,86,87,88,99]. When retrograde flow opposes anterograde flow during systole in the proximal LAD segment, bidirectional and nonlaminar (turbulent) coronary blood flow occurs, provoking (blood pressure-derived) high tensile stress (TS) and (flow-generated) low or oscillatory endothelial (wall) shear stress (ESS) [2,88,99,100,101,102,103,104,105]. The results of numerous in vivo studies in animal models and humans have shown that low or oscillatory ESS plays the most fundamental role in the development and progression of atherosclerosis [99,100,101,102,103,104,105,106,107,108,109,110,111,112,113,114]. Through complex mechanoreception and mechanotransduction processes, low and oscillatory ESS, which are characterized by low (0.5–1.5 N/m^2^ or 0.5–1.5 Pascal [Pa] or 0.5–15 dyne/cm^2^) and very low time-averaged values (<0.5 N/m^2^ or <0.5 Pascal [Pa] or <5 dyne/cm^2^), respectively, decrease production of atheroprotective endothelial vasodilatory agents (nitric oxide [NO] and prostacyclin), while simultaneously upregulating the expression of proatherogenic molecules, such as: (1) vasoconstrictive agent endothelin-1 (ET-1), (2) proinflammatory cytokines (vascular cell adhesion molecule-1 [VCAM-1], intercellular adhesion molecule-1 [ICAM-1]), and adhesion molecules (tumor necrosis factor-alpha [TNF-α], interleukin-1 [IL-1], and interferon-gama [IFN-γ]); (3) pro-oxidative, growth-promoting and prothrombotic factors (platelet-derived growth factor [PDGF]); and (4) matrix-degrading enzymes (matrix metalloproteinases [MMPs]) (Figure 2 and Figure 4) [99,100,101,102,103,104,105].

Numerous mechanoreceptors on the luminal endothelial surface, such as ion channels (K^+^, Ca^2+^, Na^+^, and C^−^), G-proteins, caveolae, tyrosine kinase receptors, nicotinamide adenine dinucleotide phosphate (NADPH) oxidase and xanthine oxidase (XO), plasma membrane lipid bilayer, and heparan sulfate proteoglycans, are capable of detecting and responding to local ESS stimuli [101,102,103,104,105]. Low and/or oscillatory ESS simultaneously activates mechanoreceptors and subsequent mechanotransduction via the cytoskeleton and several intracellular pathways. The ESS signals are transmitted to the basal and junctional endothelial surface, triggering integrins and a mechanosensory complex consisting of platelet endothelial cell adhesion molecule-1 (PECAM-1) and vascular endothelial growth factor receptors 2 and 3 (VEGFR2, VEGFR3), which initiate downstream signaling cascades. Activated integrins, PECAM-1, VEGFR2, and VEGFR3 activate Ras family GTPase via phosphorylation and activation of a multicomponent complex of non-receptor tyrosine kinases (FAK, c-Src, Shc, paxillin, and p130^CAS^), adaptor proteins (Grb2, Crk), and guanine nucleotide exchange factors (Sos, C3G). As a result, the mitogen-activated protein kinase (MAPK) cascade is activated, which has a key role in ESS mechanotransduction. Low and/or oscillatory ESS also activates other downstream signaling pathways, including the production of reactive oxygen species (ROS) from NADPH oxidase and XO, protein kinase C (PKC), endothelial nitric oxide synthase (eNOS), and phosphoinositide-3 kinase (PI3K)-Akt cascade. At the end, several transcription factors are activated, including nuclear factor-kappa ß (NF-kß) and activator protein-1 (AP-1), which bind shear stress-responsive elements (SSREs) at promoters of mechanosensitive genes, expressing proatherogenic genes and suppressing atheroprotective ones. Therefore, endothelial cells are able to convert mechanical frictional force produced by coronary blood flow into biochemical signals that regulate gene expression and cell behavior, using multiple specialized mechanisms and pathways [101,102,103,104,105].

When low or oscillatory ESS persist for a long period of time, proatherogenic molecules further promote: (1) the subendothelial (intimal) accumulation of low-density lipoprotein cholesterol (LDL-C) due to direct endothelial injury and endothelial barrier impairment with increased permeability; (2) local oxidative stress by oxidation of LDL-C; (3) the subendothelial (intimal) accumulation of monocytes, where they differentiate into macrophages that accumulate LDL-C and transform into foam cells; and (4) VSMC migration from media to intima, where they transform into a modified synthetic (secretory) phenotype, which produce collagen and MMPs, and proliferate [100,101,102,103,104,105]. The intimal accumulation of monocytes and LDL-C, along with VSMC migration and proliferation, and matrix remodeling, leads to atherosclerotic plaque formation and progression [100,101,102,103,104,105]. Accordingly, a large number of angiographic, IVUS, CCTA, pathoanatomical, and pathohistological studies, as well as computational fluid dynamic simulations, have confirmed that the LAD segment proximal to the MB is vulnerable to atherosclerosis [11,20,22,29,30,32,76,83,88,99,115,116,117,118,119,120,121,122,123,124,125,126,127,128,129]. These findings implicated that the presence of MB is a congenital anatomical risk factor for the development and progression of atherosclerosis and myocardial infarction (MI) in the proximal LAD segment, which was confirmed in three studies by Ishikawa et al. (Figure 2 and Figure 4) [119,124,125,130]. They have also revealed that the atherosclerotic lesion in the LAD appears within 20 to 30 mm of the proximal MB entrance [124,125,130]. There are at least two factors that contribute to future unfavorable atherosclerosis progression and its complications (plaque erosion, fissure or rupture, and thrombosis) in the LAD segment proximal to the MB: (1) MB muscle index (MMI) as a marker of contractile force exerted by overlying myocardial tissue (MB muscle), which is defined as a product of MB-thickness and MB-length; and (2) the presence of coexisting risk factors associated with coronary atherosclerosis, especially hyperlipoproteinemia (HLP), smoking, and diabetes [115,124,125,130,131]. The pathoanatomical study by Ishikawa et al. found that MB patients who developed MI caused by atherosclerotic lesions in the LAD segment proximal to the MB had a significantly higher MMI than those without MI (1997.6 vs. 1294.3, *p* = 0.039) [124]. This was primarily driven by increased MB-thickness and plausible greater compression of the intramyocardial LAD segment during systole. Additionally, in another pathoanatomical study by the same authors, plaque fissure or rupture in the proximal LAD segment occurred more frequently in patients with MB than in those without MB, particularly in MB patients with a high contractile force of the MB muscle and high MMI [130]. The angiography study by Yamada et al., which used IVUS for the identification of MB and atherosclerotic lesions, confirmed that systolic MB-compression (calculated as a decrease in external elastic membrane cross-sectional area [EEM-CSA] at systole divided by EEM-CSA at end-diastole, and expressed as a percentage), but not MB-length, was an independent predictor of the atherosclerotic plaque burden in the LAD segment proximal to the MB (defined as EEM-CSA − lumen CSA/EEM-CSA × 100) [115]. These findings suggest that higher MMI equals greater systolic MB-compression, which enhances systolic retrograde coronary flow toward the LAD segment proximal to the MB, resulting in low or oscillatory ESS and accelerated atherosclerosis in this LAD segment [115,124,125,130]. Furthermore, two studies revealed that the presence of coexisting risk factors, especially HLP, was an independent predictor for the development and progression of atherosclerosis in the LAD segment proximal to the MB, as a result of lipid accumulation in the subendothelial (intimal) space and inflammation [115,131]. In addition, several pathohistological studies using scanning electron microscopy observed that the endothelial cells proximal to the MB were flat and polygonal in shape, and were arranged in a pavement-like pattern, indicating that the LAD intima is stressed by low or oscillatory ESS [4,6,76,100,120,121,122,125,126].

Conversely, previous studies found that the intramyocardial LAD segment is typically spared from atherosclerosis with significantly thinner intima when compared with the intima of the proximal and distal LAD segments (Figure 2 and Figure 4) [4,6,76,129,132,133,134,135]. It was also found that the intima within the bridged arterial segment consisted only of the physiological contractile phenotype of VSMCs, together with abundant interstitial spiraled collagen, and lacked foam cells and modified synthetic phenotype VSMCs [4,6,76,100,120,121,122,125,126,129,132,133,134,135,136]. Furthermore, endothelial cells in the intramyocardial LAD segment had a helical, spindle-shaped orientation along the course of laminar antegrade and unidirectional coronary flow, while this pattern of endothelial cells was lost in the LAD segment distal to the MB [4,6,76,100,120,121,122,125,126,129]. This endothelial morphology pattern indicates that the intima at the MB-site is stressed by normal or high ESS as a result of rapid coronary flow acceleration during early diastole and the “finger-tip” phenomenon [4,6,76,100,120,121,122,125,126,129]. Normal or high ESS in the intramyocardial LAD segment, which are characterized by a positive time-averaged values (1.5–7.0 N/m^2^ or 1.5–7.0 Pascal [Pa] or 15–70 dyne/cm^2^), activated transcription factors, such as Kruppel-like family 2 (KLF2) and 4 (KLF4) and nuclear factor erythroid 2-related factor (Nrf2), leading to production of atheroprotective (anti-inflammatory, antioxidative, and antithrombotic) endothelial vasodilatory agents and downregulation of the expression of proatherogenic molecules, inducing endothelial quiescence and atherosclerosis suppression (Figure 2 and Figure 4) [100,101,102,103,104,105]. Additionally, there is some evidence that lifelong cyclic systolic MB-compression could potentially prevent lipid accumulation in the intimal space, inflammation, and subsequent development of atherosclerosis at the level of the intramyocardial LAD segment [4,100,126,137].

## 5. Myocardial Bridging and Impaired Endothelial-Dependent Vasodilation with Abnormal Coronary Vasoreactivity

Although the intramyocardial LAD segment is generally protected from atherosclerosis, it was found that this arterial segment is more susceptible to epicardial coronary vasospasm as a result of endothelial-dependent vasodilation impairment and subsequent VSMC hyperactivity to systemic vasoconstrictor stimuli (Figure 2 and Figure 4) [123,138,139,140,141,142,143,144,145,146,147,148,149,150,151,152,153,154,155,156,157]. The association between MB and epicardial coronary vasospasm was first proposed by Grover and Mancini in 1984, based on their observation of coronary spasm at the bridged arterial segment during rapid atrial pacing that was reversible with intracoronary nitroglycerin [157]. This was followed by a number of studies showing that epicardial coronary vasospasm during a positive intracoronary acetylcholine provocation test occurs more frequently in patients with than in those without MB and exists throughout the whole cardiac cycle, not only during systole [148,149,150,151,152,153]. The majority of these studies found that this epicardial coronary vasospasm was predominantly focal, involving only the coronary segment within the bridged segment [148,152,153,155]. In contrast, two studies reported that diffuse epicardial coronary vasospasm was the most prevalent type, affecting two adjacent coronary segments (the intramyocardial LAD segment and the LAD segment proximal to the MB) in 84% to 95% of MB patients [150,151]. Additionally, Nam et al. found that approximately 32% of MB patients also had a multivessel vasospasm defined as significant vasospasm in at least two major epicardial coronary arteries [150]. A study by Im et al. further revealed that MB patients with positive intracoronary acetylcholine tests at the low dose of 20 µg had a higher incidence of diffuse and multivessel vasospasms than those with positive acetylcholine tests at the high dose (50–100 µg) [146]. These findings indicate significant endothelial dysfunction and extensive epicardial coronary vasospasms in these subsets of MB patients, requiring more aggressive and intensive medical therapy.

Previous observational studies have also found that the presence of MB is an independent predictor of endothelial dysfunction due to a paradoxical response to intracoronary acetylcholine with an increased risk of epicardial coronary spasm (acetylcholine-induced coronary vasospasm) [149,150,151,152,153]. The risk of acetylcholine-induced coronary vasospasm was 1.44 to 5.15 times higher among patients with MB than among those without MB [144,149,150,151,152,153]. In addition, the vascular response to endothelial-independent coronary vasodilators nitroglycerin, isosorbide-dinitrate, and papaverine was preserved in these patients [149,150,151,152,153]. Acetylcholine, an endothelial-dependent coronary vasodilator, plays an important role in the vasoreactivity of coronary arteries. A normal, functional endothelium releases NO in response to acetylcholine, resulting in relaxation of VSMCs in the coronary artery wall [158,159,160,161,162,163,164,165]. If the coronary endothelium is dysfunctional, the NO bioavailability is attenuated and, therefore, normal vasodilatory response to intracoronary acetylcholine is instead replaced by paradoxical vasoconstriction due to its direct constrictor effect on VSMCs via stimulation of muscarinic cholinergic receptors [155,156,158,159,160,161,162,163,164,165]. Previous findings indicate the presence of abnormal vascular response in the coronary artery with MB, especially in its intramyocardial segment, due to impaired endothelial-dependent vasodilation and subsequent increased sensitivity of VSMCs to acetylcholine (Figure 2 and Figure 4) [149,150,151,152,153,155,156,160,162,163,164,165]. The extent to which vasospasm contributes to ischemia compared to coronary flow alterations caused by the bridge itself remains uncertain in most patients.

However, the underlying mechanism for abnormal vascular response in the coronary artery with MB has not been fully clarified. According to several authors, this could be attributed to the simultaneous effect of three main factors: (1) the longstanding repetitive compressive–relaxing effect of MB; (2) the high local systolic intracoronary pressure in the intramyocardial arterial segment; and (3) the flow-generated high ESS in the same arterial segment [86,100,148,149,150,151,152,155,166]. The longstanding compression–relaxation effect of MB could impair endothelial function within the bridged segment due to abnormal mechanical stimulation and direct stress of the endothelium [148,149,150,152]. This theory is supported by two studies, which confirmed that MB-length, maximal percent systolic MB-compression, and MMI as a marker of contractile MB-force (MB-thickness x MB-length, mm) were independent predictors of acetylcholine-induced coronary vasospasm in the epicardial artery with MB [140,151]. Additionally, a study by Nam et al. found that patients with severe MB (maximal percent systolic MB-compression ≥ 90% DS) had a 9.0 times higher risk of acetylcholine-induced coronary vasospasm than those with mild MB (maximal percent systolic MB-compression ≤ 50%DS) [150]. These findings suggest that higher MMI accompanied by greater systolic MB-compression is not only an independent predictor of atherosclerotic development in the coronary segment proximal to the MB, as previously explained, but also an independent predictor of coronary vasospasm in the epicardial artery with MB, especially in its intramyocardial segment [115,124,125,130,140,148,150,151,152,155]. Second, Klues et al. found the systolic intracoronary pressure was significantly higher, while the diastolic intracoronary pressure was significantly lower in the intramyocardial LAD segment, compared to the LAD segments proximal and distal to the MB [86]. Furthermore, this systolic intracoronary pressure within the bridged segment was even higher than systolic aortic and LV pressure, creating a central chamber of high and overshooting systolic pressure with a high pressure in the coronary wall at the same level (high intramural pressure) [86]. This lifelong high local systolic intracoronary pressure phenomenon further induces: (1) detrimental endogenous oxidative stress and endothelial dysfunction with reduction in NO production; (2) intimal thinning; and (3) VSMC proliferation and hyperactivity with medial thickening [5,155,166,167,168]. Third, a lifelong high ESS in the intramyocardial LAD segment driven by MB-compression also increases endogenous oxidative stress and simultaneously decreases production of both endothelium-derived relaxing (NO, endothelial NO synthase) and endothelium-derived contracting factors (ET-1, angiotensin-converting enzyme) in the endothelium of the bridged segment [5,123,155,166,167,168]. Under this condition, the protective effects of high ESS could potentially be overridden, resulting in an imbalance between endothelium-derived relaxing and endothelium-derived contracting factors and uncoupling of the vascular response to intracoronary acetylcholine [5,123,155,156,160,162,166,167,168]. Therefore, impaired endothelial-dependent vasodilation in the coronary artery with MB, especially in its intramyocardial segment, is probably the consequence of a reduction in the expression of endothelial muscarinic receptor and uncoupling from their intracellular signaling pathways [5,123,155,156,160,162,166,167,168]. In turn, VSMCs are more susceptible to acetylcholine, as it directly stimulates muscarinic cholinergic receptors in these cells, causing vasoconstriction and epicardial coronary vasospasm [5,123,155,156,158,159,160,161,162,163,164,165,166,167,168].

Finally, the clinical and prognostic importance of epicardial coronary spasm in MB patients is still underestimated. It has been documented that the presence of epicardial coronary vasospasm in patients without obstructive CAD is an independent predictor of long-term cardiac events, including myocardial infarction (MI) and SCD [158,159,169,170,171]. Numerous studies, including patients with isolated-MB, found similar results [140,141,146,149,153,155]. Ishikawa et al. listed a total of 180 English-language case reports describing MB-related acute cardiac events and myocardial ischemia, MI, and SCD [172]. Rozenberg and Nepomnyashchikh reported that 61% of patients with isolated-MB on the LAD experienced SCD, and approximately 89% in those accompanied by obstructive hypertrophic cardiomyopathy [173]. Furthermore, the presence of MB has also been identified as a leading cause of SCD among young athletes and other individuals younger than 35 years of age [173,174,175,176]. According to one meta-analysis, which included seven observational studies with approximately 5500 patients, the presence of MB was an independent predictor of MI during follow-up, which ranged from 6 to 73 months (HR: 2.75; 95% CI: 1.08–7.02; *p* = 0.03) [177]. The study by Montone et al., which included both patients with suspected stable angina and patients with suspected MI and non-obstructive coronary arteries (MINOCA), found that the presence of MB was an independent predictor of MINOCA only in patients with a positive intracoronary acetylcholine test (OR: 2.91; 95% CI: 1.285–6.578; *p* = 0.010), but not in those with a negative acetylcholine test [149]. Additionally, the positive intracoronary acetylcholine test among MB patients was the only independent predictor of MINOCA as clinical presentation (OR: 4.37; 95% CI, 1.08–17.72; *p* = 0.039). Furthermore, the same authors revealed that the presence of MB (HR: 3.98; 95% CI: 1.78–8.93; *p* = 0.001) and MINOCA as clinical presentation (HR: 4.23; 95% CI: 1.56–11.48; *p* = 0.005) were the only independent predictors of long-term cardiac events (median follow-up: 22 months), which was mainly driven by recurrent and unstable angina requiring hospitalization [149]. In a study conducted by Im et al., the cumulative incidence of mortality and recurrent angina at 1-year was significantly higher in MB patients with positive intracoronary acetylcholine tests at the low dose of 20 µg compared to those with positive acetylcholine tests at the high dose (50–100 µg) [146]. Conversely, one non-invasive study found that the vast majority of MB patients who suffered unstable angina (92%) experienced a negative Ex-SE test [54]. The study found that the presence of typical/stable angina as a clinical presentation was an independent predictor of stress-induced myocardial ischemia in MB patients (OR: 8.33; 95% CI: 1.556–44.642; *p* = 0.013), but not the presence of unstable angina [54]. Findings from these observational studies implicate a significant interplay between MB and epicardial coronary spasm, confirmed by the intracoronary acetylcholine provocation test in the development of future acute cardiac events, such as MB-related MI and recurrent and unstable angina. Consequently, more aggressive medical therapy and close clinical follow-up for MB patients with positive intracoronary acetylcholine tests, especially at the low dose of 20 µg, will probably be required to prevent future cardiac events.

## 6. Myocardial Bridging and Coronary Microvascular Dysfunction

Patients with isolated-MB may also suffer from angina and/or myocardial ischemia (ANOCA/INOCA) as a result of concomitant structural and/or functional abnormalities of the coronary microcirculation (Figure 2) [80,178,179]. Current guidelines recognize two CMD endotypes that may occur separately or synergistically: (1) structural microvascular remodeling characterized by impaired endothelium-independent vasodilation of intramyocardial arterioles as a consequence of lumen narrowing due to intimal and medial VSMC hypertrophy, perivascular fibrosis, and capillary rarefaction; and (2) functional arteriolar dysregulation characterized by impaired endothelium-dependent vasodilation and paradoxical vasoconstriction of intramyocardial arterioles (microvascular spasm) in response to intracoronary acetylcholine [80,178,179]. Studies have shown that patients with MB are at high risk for CMD (between 22% and 60%) [139,140,143,144,149,180,181]. A recent study by Allan et al. found that MB patients are more likely to experience endothelium-independent CMD, microvascular spasms, and epicardial vasospasms, which occur in 60%, 29%, and 37% of cases, respectively, with 77% showing at least one abnormality [139]. Accordingly, there is a possibility that MB could affect microvascular function remotely because endothelial dysfunction itself is associated with altered bioavailability of vasoactive agents, such as NO synthase and ET-1. Notably, CMD may be either a consequence of MB-related endothelial dysfunction or an independent coexisting abnormality. The causal relationship between MB and CMD remains unclear, and further research is required.

## 7. Future Perspectives in the Functional Evaluation of Myocardial Bridging with Implications for Therapeutic Strategies

**Figure 5:**
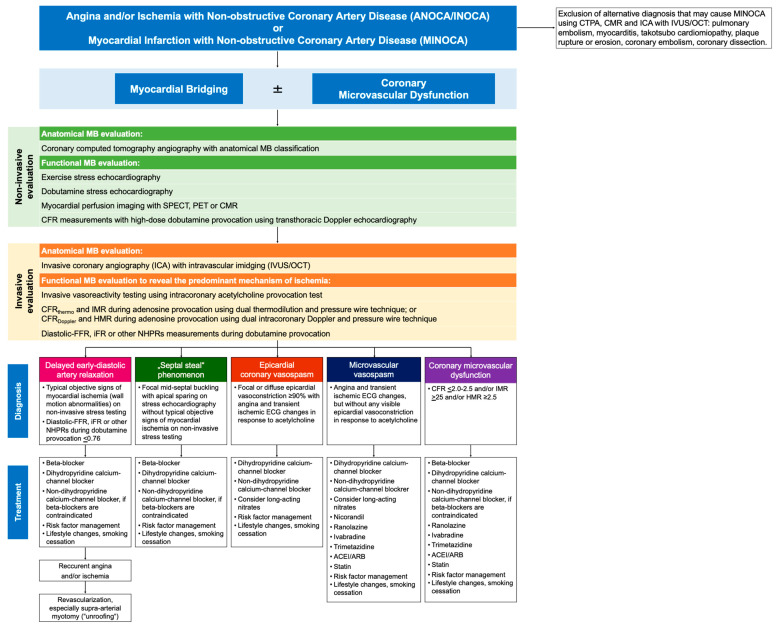
Comprehensive diagnostic and therapeutic algorithm for patients with myocardial bridging (MB). Patients with MB may have more than one phenotype, thus necessitating comprehensive non-invasive and invasive testing. Abbreviations: ACEI—angiotensin-converting enzyme inhibitors; ANOCA/INOCA—angina and/or ischemia with non-obstructive coronary artery disease; ARB—angiotensin receptor blockade; CBF—coronary blood flow; CFR—coronary flow reserve; CMR—cardiac magnetic resonance imaging; CTPA—computed tomography pulmonary angiography; IVUS—intravascular ultrasound; FFR—fractional flow reserve; HMR—hyperemic microcirculatory resistance; iFR—instantaneous wave-free ratio; IMR—index of microcirculatory resistance; MINOCA—myocardial infarction with non-obstructive coronary artery disease; OCT—optical coherent tomography; and NHPR—non-hyperemic pressure ratio.

In patients who present with ANOCA/INOCA or MINOCA and the MB on LAD revealed by CCTA or ICA, a comprehensive coronary physiology assessment should be encouraged to determine the underlying cause of angina and/or myocardial ischemia (Figure 5). Current evidence does not support the use of any physiologic index for the MB functional assessment as a stand-alone definitive test. To determine whether delayed early diastolic relaxation of the intramyocardial arterial segment is the dominant pathophysiological mechanism of angina and/or myocardial ischemia in MB patients, it is generally recommended to perform non-invasive provocation tests, such as exercise or dobutamine stress echocardiography test [2,8,9,10,54,55,82,83,84,85,86,87,88]. The characteristic echo findings are reversible wall motion abnormalities (VMA) of medial and apical segments of the anterior septum and/or anterior wall during exertion [8,54]. To detect functionally significant MB, myocardial perfusion scintigraphy is also used, which includes two types of imaging: single-photon emission computed tomography (SPECT) and positron emission tomography (PET) [182,183]. More recently, stress magnetic resonance imaging and CFR measurements assessed by transthoracic Doppler echocardiography (TTDE) have also been used for MB functional assessment. These tests require dobutamine provocation in patients with MB, which has been proven to be at least 22 times more effective at identifying functionally significant MB than adenosine, the main vasoactive agent used for evaluating the functional significance of fixed coronary stenosis [8,9,10,54,81,184,185,186,187,188]. Furthermore, it has been shown that the cut-off value of TTDE-CFR ≤ 2.1 obtained during high-dose dobutamine provocation (10–50 µg/kg/min) has the highest sensitivity, specificity, positive and negative predictive value, and diagnostic accuracy for identifying MB associated with stress-induced myocardial ischemia (96%, 95%, 88%, 98%, and 95%, respectively) [189].

For invasive physiological evaluation of MB, high-dose dobutamine provocation (>20 µg/kg/min) should be used instead of adenosine by measuring those indices that exclude the systolic phase of the cardiac cycle and the systolic pressure gradient’s impact on overall pressure measurements [8,9,10]. Diastolic-FFR is the optimal physiological index for assessing MB severity since its diagnostic accuracy is well-established [8,10]. An invasive hemodynamic study comparing conventional fractional flow reserve (FFR) and d-FFR obtained during both adenosine and dobutamine provocation against the exercise stress echocardiography as a reference in MB patients confirmed that dobutamine should be the agent of choice for the functional assessment of MB [8]. This study also showed that d-FFR obtained during inotropic stimulation with high-dose dobutamine (d-FFR_DOB_) was the only invasive test that had the ability to identify MB associated with inducible ischemia according to ischemic threshold ≤ 0.76, with a sensitivity, specificity, and positive and negative predictive value of 96%, 95%, 88% and 98%, respectively. Additionally, several invasive hemodynamic studies demonstrated that conventional-FFR during dobutamine provocation failed to identify patients with functionally significant MB due to the presence of the so-called “overshooting” effect [8,9,10,86]. This effect occurs when the average intracoronary pressure distal to the MB exceeds the average aortic blood pressure during systole (s-Pd > s-Pa), resulting in a decrease and negativization of systolic pressure gradient across the MB during inotropic stimulation. In these studies, the “overshooting” effect may artificially increase conventional-FFR values that may even exceed 1.0 (FFR-paradox) [8,9,10,86]. Other diastolic non-hyperemic (“adenosine-free”) pressure ratios (NHPR) include: (1) instantaneous wave-free ratio (iFR; Volcano Corporation, San Diego, CA, USA); (2) diastolic hyperemia-free ratio (DFR; Boston Scientific Corporation, Marlborough, MA, USA); and (3) diastolic pressure ratio (dPR; Opsens Medical, Quebec, QC, Canada), and nondiastolic NHPR—resting full-cycle ratio (RFR; Abbott Vascular, Chicago, IL, USA), which are yet to be determined against d-FFR during dobutamine provocation and/or non-invasive stress tests for the functional MB assessment [9,190].

A focal (septal) ischemia is characterized by focal mid-septal buckling with apical sparing during end-systole to early diastole on exercise stress echocardiography [95,191]. Overall diagnostic accuracy of this echo pattern in identifying the presence of MB in the LAD by CCTA was 89%, with a sensitivity, specificity, and positive and negative predictive value of 90%, 83%, 96.5% and 62.5%, respectively [191]. Although the presence of focal mid-septal buckling with apical sparing on exercise stress echocardiography is a reliable predictor of MB existence in patients with angina and non-obstructive CAD, it does not necessarily indicate its functional importance [191]. Based on current guidelines, it seems that this focal septal WMA on exercise stress echocardiography indicates the existence of altered intracoronary hemodynamics in the LAD with MB, rather than functionally significant MB.

In MB patients with suspected ANOCA/INOCA or MINOCA and non-obstructive CAD, a comprehensive “invasive coronary functional testing” (ICFT) is essential not only to confirm MB-related ischemia, but also to identify alternative or additional mechanisms of symptoms (Figure 5) [78,79]. This ICTF should include: (1) MB testing with intravascular imaging (intravascular ultrasound, optical coherent tomography); (2) invasive vasoreactivity testing using intracoronary acetylcholine provocation test to evaluate epicardial and microvascular vasospasm, followed by intracoronary nitroglycerin; (3) invasive CFR and index of microcirculatory resistance during adenosine provocation using dual thermodilution and pressure wire technique (CFR_thermo_ and IMR, respectively), or CFR and hyperemic microcirculatory resistance during adenosine provocation using dual intracoronary Doppler and pressure wire technique (CFR_Doppler_ and HMR, respectively), for assessing CMD; and (4) d-FFR or other NHPRs measurements during high-dose dobutamine provocation to evaluate the functional MB significance [78,79,186]. As a result of this invasive testing, it may be possible to identify the predominant mechanism of ANOCA/INOCA or MINOCA in MB patients, leading to a more effective therapeutic approach.

An intracoronary acetylcholine provocation test is the gold standard for diagnosing coronary vasomotor disorders (epicardial endothelial dysfunction, epicardial and microvascular vasospasms) [78,79,80,178,186,192]. It is generally performed without intracoronary wire before adenosine infusion because intracoronary nitroglycerin (200 µg) is routinely recommended after acetylcholine testing to resolve epicardial vasospasms and to confirm that epicardial endothelium-independent vasodilation has been achieved before adenosine-induced maximal hyperemia [79]. In this manner, intracoronary nitroglycerin is avoided immediately before acetylcholine testing, which may mask acetylcholine-induced coronary vasospasm. An ergonovine provocation test is an appropriate alternative in identifying coronary vasospasm in patients with suspected ANOCA/INOCA or MINOCA, especially in those with negative acetylcholine testing [192,193,194,195]. However, there are no data regarding its accuracy in identifying coronary vasospasm in patients with MB.

An acetylcholine provocation test is considered positive for epicardial coronary vasospasm and vasospastic angina (VSA) if all of the following are present in response to vasoactive stimuli: (1) transient focal or diffuse epicardial coronary diameter reduction ≥ 90% assessed by QCA compared with the resting condition; (2) transient ischemic ECG changes (ST-elevation ≥ 0.1 mV, ST-depression ≥ 0.1 mV, or new appearance of negative U waves), recorded in at least two contiguous leads; and (3) clinical onset of angina or angina equivalents (i.e., shortness of breath) [78,79,80,178,192,196,197,198,199,200,201,202,203,204,205,206]. Vasospastic angina is the clinical manifestation of myocardial ischemia caused by epicardial coronary vasospasm, and the international diagnostic criteria of both VSA and epicardial coronary vasospasm were proposed by the Coronary Vasomotion Disorders International Study (COVADIS) group in 2015 [207]. Epicardial endothelial dysfunction is currently defined as any epicardial vasoconstriction of >0% but <90% after low-dose acetylcholine provocation as determined by QCA [79,80,178]. An acetylcholine provocation test is considered positive for microvascular vasospasm and microvascular angina if angina or angina-like symptoms and typical transient ischemic ECG changes occur, but without any visible epicardial vasoconstriction in response to acetylcholine [78,79,80,140,178,181,192,208,209,210,211,212]. It may also be evaluated by measuring volumetric coronary blood flow (CBF) and CFR using a Doppler-tipped guidewire and quantitative coronary angiography during acetylcholine provocation [208,209,210,211,212]. Microvascular angina is the clinical manifestation of myocardial ischemia caused by microvascular vasospasm, and the international COVADIS diagnostic criteria of both MVA and microvascular vasospasm were proposed in 2018 [213].

Finally, in patients with angina and non-obstructive CAD, a CFR threshold of less than 2.0 to 2.5, depending on the diagnostic modality, is used as a diagnostic criterion for the presence of both CMD endotypes [78,79,80,178]. The reduced CFR below ischemic threshold, accompanied by an increased IMR ≥ 25 and/or HMR ≥ 2.5 mmHg/cm) in response to endothelium-independent vasodilatory agents such as adenosine or papaverine, as a result of extensive myocardial fibrosis and subsequent pathologically increased minimal hyperemic microvascular resistance, indicates the presence of structural CMD [78,79,80,178,186,196,197]. A reduced CFR below ischemic threshold with normal IMR or HMR indicates the presence of functional CMD. A low CFR in functional CMD could be the result of high resting coronary flow due to increased oxygen demand or abnormal metabolic state and, therefore, requires further evaluation with an intracoronary acetylcholine provocation test to determine the definitive mechanism of angina and/or ischemia in patients with non-obstructive CAD [78,79,80,178,186,196,197]. Recently, two novel markers have been proposed that represent the vasodilatory capacity of coronary microcirculation: the resistive reserve ratio (RRR) and the microvascular resistance reserve (MRR), whose diagnostic significance is yet to be determined in the evaluation of CMD [78,79,198,199,200,201,202,203,204,205,206].

Empirical medical therapy using different antianginal medications is the first-line strategy for treatment of MB patients (Figure 5). Beta-blockers, with or without dihydropyridine calcium-channel blockers (DHP-CCBs), have been generally recommended as the first-line therapy in asymptomatic MB patients with documented altered intracoronary hemodynamics, as well as in symptomatic MB patients with documented objective signs of myocardial ischemia. Non-dihydropyridine calcium-channel blockers (nonDHP-CCBs) can be used as an alternative to beta-blockers in those MB patients who are unable to tolerate or have contraindications to beta-blockers. A combination of DHP-CCB and nonDHP-CCB is the recommended first-line therapy in MB patients with documented acetylcholine-induced epicardial and microvascular vasospasm, since beta-blockers are contraindicated. Long-acting nitrates should be avoided in these patients at present since their efficacy and safety in MB patients are still unclear. In addition, lifestyle changes (exercise training, weight loss, and smoking cessation) and management of risk factors (hypertension, dyslipidemia, metabolic syndrome, and diabetes) have an important role in improving anginal symptoms and preventing progression of epicardial and microvascular changes (Figure 5). Therefore, the use of antianginal, atheroprotective, and anti-inflammatory agents, such as angiotensin-converting enzyme inhibitors or angiotensin receptor blockade, beta-blockers, CCBs, statins, ranolazine, trimetazidine, nicorandil, and ivabradine, is beneficial in patients with non-obstructive CAD [79,80,214].

A supra-arterial myotomy (“myocardial unroofing”) is a favorable surgical procedure and has an advantage over MB-stenting in symptomatic MB patients with angina and stress-induced myocardial ischemia not responding to optimal medical therapy. MB-stenting is not suitable since it is associated with adverse events, especially coronary perforation during a procedure and in-stent restenosis at long-term follow-up. In patients with MB and functionally significant fixed coronary stenosis in the LAD segment proximal to the MB who are not eligible for PCI, coronary artery bypass grafting is a favorable surgical option. Additional studies are required to improve MB patient stratification according to the underlying mechanism of angina and/or myocardial ischemia and specific MB phenotype, and to define optimal therapeutic strategies in these patients. A detailed description of diagnostic and therapeutic algorithms for MB patients can be found elsewhere [215].

## 8. Conclusions

Myocardial bridging (MB) may cause different forms of ischemic heart disease, which could be associated with life-threatening ventricular arrhythmias, LV systolic dysfunction, or even SCD. The growing body of evidence emphasizes four different pathophysiological mechanisms of MB-related angina and/or myocardial ischemia that may act independently or synergistically. Patients with isolated-MB may also have anginal symptoms and/or myocardial ischemia due to concomitant structural or functional CMD. Therefore, comprehensive coronary physiology testing should be encouraged in patients with this coronary anomaly to identify the underlying cause of anginal pain and/or myocardial ischemia, enabling optimal therapeutic strategies in these patients.

## Figures and Tables

**Table 1:** Contemporary anatomical classification of myocardial bridging (MB) on the left anterior descending artery (LAD) based on coronary computed tomography angiography (CCTA) findings.

MB Type	The Superficial Type	The Deep (Intramural) Type	The Right Ventricular Type
**MB-depth**	Depth of the intramyocardial LAD segment ≤ 1.0 mm from the anterior interventricular groove surface.	Depth of the intramyocardial LAD segment > 1.0 mm from the anterior interventricular groove surface.	Depth of the intramyocardial LAD segment > 1.0 mm from the anterior interventricular groove surface.
**MB-course**	Superficial course of the intramyocardial LAD segment along the interventricular septum.	The intramuscular LAD segment penetrated the interventricular septum, approaching the right ventricular anterior wall.	The intramuscular LAD segment crossed through the right ventricular anterior wall or within the right ventricular cavity, adjacent to the interventricular septum.
**Overlying myocardial tissue**	Covered by a thin layer of tissue.	Covered by a thick layer of tissue.	Covered by a thick layer of tissue.

The intramyocardial LAD segment tends to deviate toward the right ventricular aspect of the interventricular septum in its deeper segments. The deepest MB forms could pass through the right ventricle’s anterior wall adjacent to the interventricular septum or even through its cavity.

## Data Availability

No new data were created or analyzed in this study.
